# Multiple paternity in the freshwater snail, *Potamopyrgus antipodarum*

**DOI:** 10.1002/ece3.408

**Published:** 2012-11-16

**Authors:** Deanna M Soper, Lynda F Delph, Curt M Lively

**Affiliations:** Department of Biology, Indiana University1001 E. Third St., Bloomington, Indiana, 47405

**Keywords:** Genetic diversity, multiple paternity, polyandry, sire evenness

## Abstract

Mating multiply may incur costs, such as exposure to predators and to sexually transmitted diseases. Nevertheless, it may be favored, in spite of these costs, as a way to increase the genetic diversity of offspring through fertilization by multiple males. Here, we tested for multiple paternity in a freshwater snail (*Potamopyrgus antipodarum*), which is host to several species of sterilizing trematode worms. Using microsatellites markers, we found multiple paternity in two different snail populations, with as many as seven males fertilizing a single female. In addition, high evenness of sire fertilization was found within individual broods. Multiple paternity can occur for a variety of reasons; however, given that these populations experience high risk of infection by a sterilizing trematode, one potential explanation may be that multiple paternity and high evenness of sire fertilizations increase the chances of the production of parasite-resistant offspring.

## Introduction

Multiple paternity, wherein females mate with and are fertilized by more than one male, occurs in a wide variety of plant and animal species (Teixeira and Bernasconi 2007; Uller and Olsson 2008). This phenomenon can be selected for, or against, depending on the various costs and benefits of mating present in a given system (Ala-Honkola et al. 2011; House et al. 2011). For example, polyandry and polygamy may be costly because mating happens more than once, potentially causing increased exposure to sexually transmitted diseases (Thrall et al. 1997, 2000) or predators (Magnhagen 1991; Arnqvist 1997). Moreover, mating has been shown to decrease female life span or fecundity in animals as a consequence of increased exposure to sperm accessory proteins (Wing 1988; Chapman et al. 1995; Orsetti and Rutowski 2003), decreased time for resource allocation (Bowcock et al. 2009), and increased risk of harm from males (Sakurai and Kasuya 2008; den Hollander and Gwynne 2009).

Despite the possible costs, the widespread nature of polyandry that results in multiple paternity suggests that the benefits of mating multiply may outweigh the costs. Many hypotheses have been put forward, including some that address how females may benefit from multiple paternity. For example, females might be selected to mate with multiple males to ensure viable offspring in populations that commonly experience genetic incompatibility between males and females (Zeh and Zeh 1996, 1997). In addition, multiple paternity may be favored as a way to increase the genetic diversity among offspring within and/or between broods (Yasui 1998; McLeod and Marshall 2009). The benefit of increasing the genetic variability among offspring has been shown theoretically to apply to systems in which parasites significantly reduce host fitness (Tarpy 2003; Hughes and Boomsma 2004; Seeley and Tarpy 2007).

**Figure 1:**
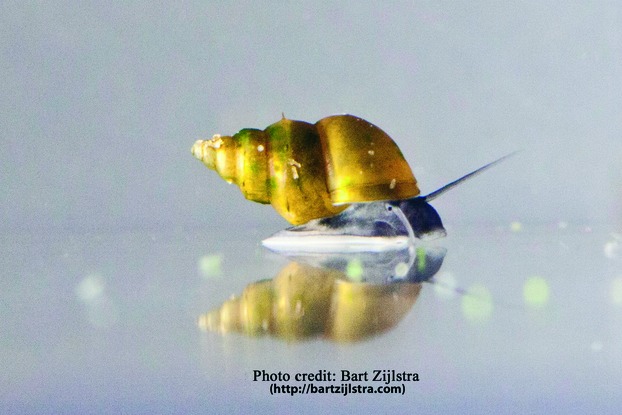
*Potamopyrgus antipodarum*; photo credit: Bart Zijlstra; http://bartzijlstra.com.

Here, we investigate whether multiple paternity exists in *Potamopyrgus antipodarum*, a freshwater snail that is native to New Zealand (Fig. 1). The snail is host to over a dozen digenetic trematodes, several of which sterilize infected individuals of both sexes (Lively 1987; Jokela and Lively 1995; King and Lively 2009). Moreover, the most common parasite, *Microphallus* sp., has been shown to be able to track and over infect common genotypes (Dybdahl and Lively 1995; Jokela et al. 2009; Koskella and Lively 2009). Third, females exposed to parasites mate more frequently and with an increased number of males than unexposed female (King, Soper, Vergara, and Lively, unpubl. data). Finally, both sexual and asexual females exist, and the prevalence of sex is positively correlated with the prevalence of the parasitic trematodes (Lively 1992; Lively and Jokela 2002). Consequently, we predicted that the broods of sexual females would be multiply sired, and we tested this prediction using snails from two lakes containing relatively high proportions of sexual females and trematode infections (Lively 1987; Lively and Jokela 2002; Jokela et al. 2009).

## Methods

### Study system

*Potamopyrgus antipodarum* is a small snail (2.0–5.0 mm) that is endemic to lakes and streams in New Zealand (Winterbourn 1970). Populations of *P. antipodarum* are characterized by diploid, sexual males and females and/or polyploid, parthenogenetic females (Fox et al. 1996; Neiman et al. 2011). *Potamopyrgus antipodarum* undergoes internal fertilization, gestation, and live birth (Winterbourn 1970).

### Sampling and microsatellite analysis

Female *P. antipodarum* were randomly sampled from the shallow-water margins of Lake Alexandrina and Lake Kaniere on the South Island of New Zealand in January 2010. The snails were flash frozen in liquid nitrogen, shipped to Indiana University, Bloomington, and placed in a −80°C freezer. Females were dissected and somatic tissue from the head was isolated for flow-cytometry to determine which females were sexual (diploid) versus asexual (triploid). Broods and one tentacle from each female were placed individually in microcentrifuge tubes, flash frozen, and stored in a −80°C freezer until ploidy could be determined. Once sexual (diploid) females were identified, the brood sac for each sexual female was dissected and each embryo was isolated into a single microcentrifuge tube. The DNA was extracted using a Chelex procedure and amplified using polymerase chain reaction (PCR) using three different microsatellite primers. A 3730 DNA analyzer at the Indiana University Molecular Biology Institute used GeneScan (Seeb et al. 2000) to analyze amplified DNA samples. Raw output from the 3730 DNA analyzer was read using GeneMapper 4.1 (Applied Biosystems, Carlsbad, CA, USA).

### Flow-cytometry methods

Each sample was ground with 100-μL cold dimethyl sulfoxide (DMSO) buffer (0.25 mol/L sucrose, 40 mmol/L trisodium citrate dehydrate, 0.5% DMSO). The cell suspension was stained with 750 μL of a propidium idodide solution containing spermine (20-mg propidium iodide and 116-mg spermine tetrahydrochloride) in 100-mL detergent stock solution: 3.4-mmol/L trisodium citrate dehydrate, 0.1% Nonidet p-40, 1.5-mmol/L spermine tetrahydrochloride, and 0.5-mmol/L Tris) (Osnas and Lively 2006). This stain attaches to the DNA molecule and allows the flow cytometer to determine the amount of DNA content using a laser and measurement of refraction. The sample was filtered using Partec 50-μm CellTric filters and placed on ice for <2 h until the samples could be run on a FACScan flow cytometer (Becton-Dickinson, Franklin Lakes, NJ, USA). Two standards were prepared in an identical manner. One standard was from a known diploid lineage and the other from a known triploid linage. Standards allow for comparison of experimental samples, which results in the ability to detect whether experimental samples are diploid or triploid.

### DNA-extraction methods

DNA was extracted from each embryo and a tentacle from each female using Chelex. Each sample was ground in 150 μL of 5% Chelex using a motorized pestle and incubated at 56°C for 45 min. After incubation, the samples were vortexed and centrifuged before being incubated at 98°C for 20 min. The samples were then centrifuged again, which separated the DNA into the upper layer. The DNA was removed using a pipette, and moved to a new, sterile, labeled microcentrifuge tube. The extracted DNA was stored frozen until PCR could be accomplished.

### PCR methods

A reagent mix of ddH_2_O, Titanium Taq DNA Polymerase (Clontech, Mountain View, CA, USA), Titanium Taq Buffer (Clontech), dNTP, forward primer, reverse primer, and M13 (a florescent primer) was made. The solution contained 11.5 μL ddH_2_O, 2.5 μL of Titanium Taq buffer, 0.1 μL of forward primer, 0.2 μL of reverse primer, 0.2 μL of M13, 0.5 μL of dNTP, and 0.5 μL of Titanium Taq DNA Polymerase. DNA (10 μL) from each sample was transferred to a well and a diagram of the location of the samples was recorded. The reagent mix (15.5 μL) was added to each well. Three different primers were used to amplify each DNA sample (Weetman et al. 2002). The microsatellite sequences used are as follows: Primer #1, Pa143 – F: 5′ TGT CGT GTG TCA AAT ACA CAT TAT 3′ and R: 5′ GAG CTC ACT GGA GGA AAA GC 3′; Primer #2, Pa 254 – F: 5′ CCC TTT CAT TTG CAG AGA GC 3′ and R: 5′ GTC GTC AAA ACC CCT GTA CG 3′; Primer #3, Pa 121 – F: 5′ GGA AAA GCG CGT TTA AGC ATC 3′ and R: 5′ TTG CGC CAC AGA GCC AAG C 3′. PCR reactions for primers Pa143 and Pa121 consisted of denaturing at 95°C for 1 min and then 35 cycles of 95°C for 30 sec and 68°C for 3 min followed by 68°C for 3 min. PCR reactions for primer Pa254 denatured at 94°C for 2 min and then a step-down procedure in two-degree increments starting at 61°C for 1 min then 72°C for 1 min for 27 cycles and then ending with a cycle of 72°C for 5 min.

After the DNA was amplified, it was prepared for analysis using the Applied Biosystems 3730 DNA analyzer. Amplified DNA was diluted in a 1:60 ratio before being transferred to an ABI plate. The ABI plate is a skirted 96-well plate used for analysis within the DNA analyzer. Using a multichannel pipette, 1 μL of diluted PCR product was added to the ABI plate. A ladder mix was created by adding 17 μL of Liz ladder (Applied Biosystems) to 983 μL of ddH_2_O. Each sample will have 9 μL of this mix added to the well already containing the PCR product. The ABI plate was vortexed and centrifuged before being placed in the 3730 DNA analyzer.

### Parternity analysis

Microsatellites were read using GeneMapper (Applied Biosystems). Peaks that were within regions of known amplification (Weetman et al. 2002) and which were consistent within the population were identified and recorded for each sample. A new round of PCR and DNA analysis was conducted for samples that had ambiguous or no peaks. Samples that consistently failed DNA amplification were removed from the data set. Removal resulted in a reduction in brood size in the final data set, but may have yielded more conservative estimates of multiple paternity.

The allele sets for each mother and brood were analyzed using allele counting (Simmons et al. 2008), GERUD (Jones 2005), and COLONY (Wang 2004; Wang and Santure 2009). To accomplish allele counting, we recorded the paternal alleles, identified the most diverse locus, and divided the number of alleles by 2. The result is the estimated number of sires for that brood. This method is conservative because it assumes that each sire is heterozygous at that locus. GERUD is a computer program that estimates the minimum number of fathers even when the father genotypes remain unknown. GERUD starts by subtracting the known maternal genotype and then determines the minimum number of sires that can explain the entire progeny array. If multiple solutions exist, the program can then determine the most likely solution based on the laws of Mendelian segregation. The program assumes that all offspring are full or half-siblings. This is true within the snails because there is internal fertilization and broods were dissected from the female body. Therefore, all offspring are at least half-sib, all sharing the same mother. COLONY is also a computer program that uses mathematical algorithms to determine the number of sires when their genotypes remain unknown. Although GERUD estimates the minimum number of sires, COLONY estimates the most likely number of sires. Thus, COLONY was used to estimate the maximum number of sires per brood.

### Sire evenness

In addition to estimating the most likely minimum number of sires for each brood, GERUD 2.0 (Jones 2005) calculates the most likely distribution of offspring across sires. We used these proportions for observed frequencies of sire evenness. Sire evenness was calculated in the same manner as (Schmoll et al. 2007), where a sire diversity index *D*, based on the Shannon–Wiener Index, is utilized to calculated sire evenness *E*. Sire diversity *D* is calculated by (following Schmoll et al. 2007):


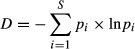


In this formula, *S* equals the number of genetic sires in a brood and *p*_*i*_ equals proportion of offspring sired by the *i*^th^ sire. Once *D* was obtained, evenness *E* could be calculated by utilizing an additional index, *D*_*max*_. This index is a hypothetical diversity score where *D*_*max*_ = ln *S* and thus this value is the maximum possible sire diversity if offspring had been distributed evenly given the number of genetic sires. The amount to which a female has reached maximum diversity within the clutch if limited to a set number of sires (evenness) is calculated by (following Schmoll et al. 2007):


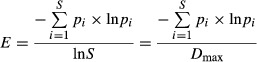


Four broods had ambiguous paternity of at least one offspring and as a result these broods are excluded from the evaluation of evenness.

### Statistical analysis

To determine whether sire evenness deviated from expected, Generalized Estimating Equations were used in SPSS, version 19. The model compared the distribution of sire evenness scores with that of a normal linear distribution. If significantly different, the sire evenness can be concluded as being nonrandom. Values close to 1 indicate a highly even brood, thus if values are high and nonrandom the sire evenness can be thought of as being greater than expected by chance.

## Results

**Figure 2:**
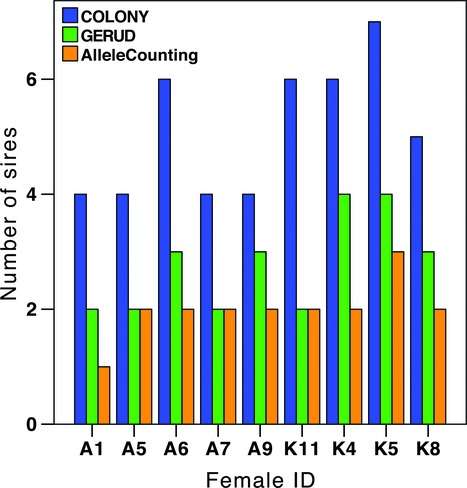
Estimated number of sires for each female using, COLONY (in blue), GERUD (in green), and allele counting (orange).

Five sexual females were analyzed from Lake Alexandrina and four from Lake Kaniere. Females from Lake Alexandrina had broods that ranged in size from 5 to 13 embryos, whereas females from Lake Kaniere had broods that ranged from 12 to 20 embryos (Table 1). Allele counting, a conservative estimate, found multiple paternity in eight of nine broods (Fig. 2). Using the allele-counting method, females from Lake Alexandrina had broods that were fertilized by 1–2 sires, and females from Lake Kaniere had broods that were fertilized by 2–3 sires. GERUD 2.0 was also used to estimate multiple paternity. Using this method, we found multiple paternity for the broods of every female from both lakes (Fig. 2). Using GERUD, broods from Lake Alexandrina females were estimated to have 2–3 sires and broods from Lake Kaniere females 2–4 sires. COLONY estimated number of sires higher than GERUD for each female (Table 1). Lake Alexandrina broods were estimated to have 4–6 sires and broods from Lake Kaniere were estimated to have 5–7 sires (Fig. 2).

**Table 1:** Each sexual female is listed with number of embryos analyzed and estimated number of sires using COLONY, GERUD, and allele counting

Population	Female ID	Number of analyzed embryos	Number of sires using COLONY	Number of sires using GERUD	Number of sires using allele count
Alexandrina	A1	8	4	2	1
Alexandrina	A5	6	4	2	2
Alexandrina	A6	13	6	3	2
Alexandrina	A7	5	4	2	2
Alexandrina	A9	6	4	3	2
Kaniere	K4	12	6	4	2
Kaniere	K5	16	7	4	3
Kaniere	K8	19	5	3	2
Kaniere	K11	20	6	2	2

Sire evenness, a measure of the proportion of fertilization by a given sire within a brood, was analyzed. Because GERUD estimates the entire data set taken together, some broods may have one or more embryos that are compatible with more than one possible sire. When this occurs the paternity of the offspring is said to be ambiguous. Using GERUD, we found that four of the nine broods had ambiguous paternity. To ensure we did not over estimate evenness, we used only the broods whose paternity was not ambiguous (five of nine broods). Sire evenness was found to range from 0.81 to 0.97 (Table 2). Because a value of 1 would yield a completely even brood, our broods were found to be high in evenness, but were significantly different from 1 (95% CI = 0.833–0.987), and also different from random (GEE: *N* = 5; *P* ≤ 0.001).

**Table 2:** Calculated sire evenness for each female

Population	Female ID	Sire evenness
Alexandrina	A1	0.81
Alexandrina	A7	0.97
Alexandrina	A9	0.92
Kaniere	K5	0.95
Kaniere	K8	0.90

## Discussion

The aim of this study was to document whether multiple paternity occurs in *P. antipodarum*. We predicted that sexual females of the freshwater snail *P. antipodarum* would exhibit multiple paternity within their broods. While we did not address the causal mechanism for why females mate multiply, there may be many reasons, including the generation of genetic diversity. If sex is maintained in *P. antipodarum* as a consequence of it increasing the genetic diversity of offspring and thereby avoiding tracking by the parasite, then multiple paternity would take this one step further: it would increase the genetic diversity of a brood even more than producing offspring with a single male. We based this prediction on the fact that the trematode parasites of this snail are able to track common host genotypes, and that they are highly virulent, effectively sterilizing any infected snail host (Lively 1987, 1989; Dybdahl and Lively 1995, 1998; Jokela et al. 2009). Multiple paternity could increase the genetic diversity of sexually produced offspring, and potentially increase the fitness of females adopting this mating strategy.

We used allele counting, GERUD 2.0, and COLONY to determine whether multiple paternity existed. Allele counting is the most conservative method, because it only takes into account paternity at the most diverse locus by dividing the number different alleles by two. Because each potential set of parents contributes no more than two alleles, the number of sires cannot be lower than this estimate. This method may underestimate the number of sires, as it does not take into account the entire progeny array. Thus, we also used two computer programs: GERUD, which estimates the minimum number of sires after examining the entire progeny array (Jones 2005) and COLONY, which estimates the most probable number of fathers (Wang 2004).

GERUD 2.0 found multiple paternity in the broods of all nine sexual females examined; the number of estimated sires ranged from 2 to 4. In addition, COLONY found multiple paternity in all nine broods, with an estimated sire range of 4–7. Our prediction of multiple paternity was strongly supported by the data. However, the study was limited in scope. Additional studies may benefit from inclusion of marker polymorphism data within parentage reconstruction, as it would provide more exact estimates of sire number and evenness. In addition, increasing sample size of females and number of lakes would verify the pervasiveness of the occurrence of multiple paternity within and across populations.

Sire evenness within each brood was high, which would have contributed to maximizing the genetic diversity of the broods. Several mechanisms could have contributed to this evenness, ranging from the simple mixing of sperm from multiple males, to more active mechanisms by the females, such as choice of which sperm to use in fertilization or which embryos to develop. For example, in the plant *Oenothera organensis*, the proportion of ovules fertilized by each sire did not fit the proportion of seeds developed, as the plant aborted embryos in a way that increased the evenness of the siring (Havens and Delph 1996). In animals, bias in siring success can correlate with mating order, where either the first or last mating male sires a larger portion of offspring (Smith 1979; Austad 1982). In the pseudoscorpion, *Cordylochernes scorpioides*, last male precedence occurs only if two males mate; however, when a female mates with a third male, equal numbers of offspring are sired by each male (Zeh and Zeh 1994). An association between sire evenness and number of sires may select for females that exhibit promiscuous mating behavior, especially in populations where genetically diverse broods may be advantageous.

Mating is thought to be costly, because it may take time away from foraging, increases exposure to predators, and increases the risk of parasitism (Emlen and Lewis 1977; Daley 1978; Sherman et al. 1988; Wing 1988; Chapman et al. 1995; Thrall et al. 1997, 2000; Bowcock et al. 2009). In *P. antipodarum*, mating snails assume an aperture-to-aperture mating position where they remain stationary for the length of mating (Soper and Lively, unpubl. data), which can last for more than an hour (Soper, pers. obs.). Mating may be costly in *P. antipodarum*, because the stationary nature of mating may increase exposure to predators and reduce foraging time.

Multiple paternity may increase genetic diversity within a brood, and thus give multiple fertilized females an advantage (Neff and Pitcher 2005; McLeod and Marshall 2009). This may be especially important in populations that experience uncertain environments (Yasui 1998), which may be caused by parasites (Fuchs and Schade 1994; Baer and Schmid-Hempel 1999; Tarpy 2003; Seeley and Tarpy 2007). While there may be many reasons for the existence of multiple paternity in *P. antipodarum*, it is possible that coevolving parasites might not only select for sex over asex, but for more promiscuous sexual behavior.
